# Effects of *Spirulina platensis* on insulin secretion, dipeptidyl peptidase IV activity and both carbohydrate digestion and absorption indicate potential as an adjunctive therapy for diabetes

**DOI:** 10.1017/S0007114520002111

**Published:** 2020-11-28

**Authors:** J. M. A. Hannan, Prawej Ansari, Shofiul Azam, Peter R. Flatt, Yasser H. A. Abdel Wahab

**Affiliations:** 1 Department of Pharmacy, Independent University, Dhaka 1229, Bangladesh; 2 School of Biomedical Sciences, Ulster University, Co. Londonderry, Northern Ireland BT52 1SA, UK; 3 Department of Integrated Bioscience, Graduate School, Konkuk University, Chungju 27478, Republic of Korea

**Keywords:** Diabetes, Plant therapies, Glucose, Insulin

## Abstract

*Spirulina platensis* has been found to be useful in the treatment of type 2 diabetes. The present study aims to elucidate the effects of ethanol extract and butanol fraction of *S. platensis* on insulin release and glucose homoeostasis in type 2 diabetic rats, together with their mechanism of actions. *In vitro* and *in vivo* methods were used including cellular studies to determine potential role of ion channels and cAMP in the insulinotropic actions of the extracts. The ethanol extract and butanol fraction stimulated insulin release from mouse islets and pancreatic *β*-cells in a concentration-dependent manner. The butanol fraction also similarly stimulated insulin release from perfused rat pancreas. The insulin-releasing action was augmented by glucose, isobutylmethylxanthine, tolbutamide and a depolarising concentration of KCl. The insulin secretory effect was attenuated with diazoxide and verapamil and by omission of extracellular Ca^2+^. Butanol fraction was found to significantly inhibit dipeptidyl peptidase IV enzyme activity. Moreover, butanol fraction improved glucose tolerance following oral glucose administration (2·5 g/kg body weight (b.w.)). The butanol fraction was tested on 24 h starved rats given an oral sucrose load (2·5 g/kg b.w.) to examine possible effects on carbohydrate digestion and absorption. *S. platensis* substantially decreased postprandial hyperglycaemia after oral sucrose load and increased unabsorbed sucrose content throughout the gut. During *in situ* intestinal perfusion with glucose, the butanol fraction reduced glucose absorption and promoted gut motility. Finally, chronic oral administration of butanol fraction for 28 d significantly decreased blood glucose, increased plasma insulin, pancreatic insulin stores, liver glycogen and improved lipid profile. The characterisation of active compounds from butanol fraction revealed the presence of *p*-coumaric acid, *β*-carotene, catechin and other antioxidant polyphenols. In conclusion, *S. platensis* could be an adjunctive therapy for the management of type 2 diabetes.

Diabetes mellitus is a metabolic syndrome where pancreatic *β*-cells fail to meet the body’s need for insulin with resultant hyperglycaemia and increased risk of diabetic complications. The WHO recognised diabetes mellitus as the world’s fastest growing metabolic disorder. Type 1 diabetes is the result of total or near-total *β*-cell destruction, whereas type 2 diabetes mellitus (T2DM) is the result of *β*-cell dysfunction and insulin resistance. About 90 % of diabetic patients suffer from T2DM. This condition is associated with the alterations in the metabolism of lipids, carbohydrates and proteins^(1)^, which causes multiple complications including CVD^(2)^, retinopathy^(3)^, neuropathy, cognitive decline^(4)^, nephropathy and end-stage renal disease^(3)^.

T2DM treatments include diet together with either a single or combination of oral anti-hyperglycaemic agents, to manage dysglycaemia^(5,6)^. Although advances have been made recently in the treatment options to achieve better glycaemic control, they are often expensive and associated with notable adverse effects^(7,8)^. This focuses attention particularly in poorer countries towards herbal therapy and dietary supplements as alternative approaches to the mainstream medical treatment of T2DM. Unlike contemporary treatment, herb-based medicines are entirely natural; they possess very few adverse effects and are generally affordable^(9)^. It has been reported that nearly 30–76 % of T2DM patients from different countries are using herbal medicines^(10,11)^ and that this approach is managing T2DM with safety^(12)^.

Over the last decades, functional foods received attention as a potential source of useful bioactive protein hydrolysates and peptides that have health benefits and reduce the risk of disease^(13)^. In this way, many aquatic species have been studied with the generation of new and useful bioactive peptide sources. For instance, the eukaryotic microalgae and prokaryotic cyanobacteria (often called blue-green algae) utilisation in the food industry has become globally popular^(14,15)^. Edible microalgal and cyanobacterial proteins have been established as suitable precursors for the production of biologically active peptides^(14)^. Thus, a series of peptides isolated from microalgal or cyanobacterial hydrolysates have been demonstrated to exhibit desirable bioactivities, such as antioxidative, anticancer, anti-inflammatory and antihypertensive properties^(13,16–18)^.

*Spirulina platensis* is a unicellular cyanobacterium belonging to the Cyanophyceae class, Oscillatoriaceae family^(19)^. This organism (cyanobacterium) is characterised by spiral chains of the cells enclosed in a thin sheath. It contains very potent naturally occurring antioxidants and free radical scavenging agents^(20)^. *S. platensis* is non-toxic, bioavailable, and is believed to provide significant multiorgan protection against many drugs and chemically induced toxic assaults^(21)^. Active constituents exhibit anti-inflammatory, neuroprotective, hepatoprotective, immunomodulatory and anticancer activities^(22)^. A recent animal study reported potential anti-diabetic effects of *S. platensis*, but the mechanisms underlying such effects are unknown^(23–25)^. In this paper, we have made a detailed study of effects of *S. platensis* on pancreatic insulin release, dipeptidyl peptidase IV (DPP-IV) inhibition and various gastrointestinal (GI) tract actions to elucidate the mechanism and therapeutic potential of *S. platensis* for improvement of diabetes control.

## Materials and methods

### Plant material and preparation of ethanol extract

**Fig. 1.**
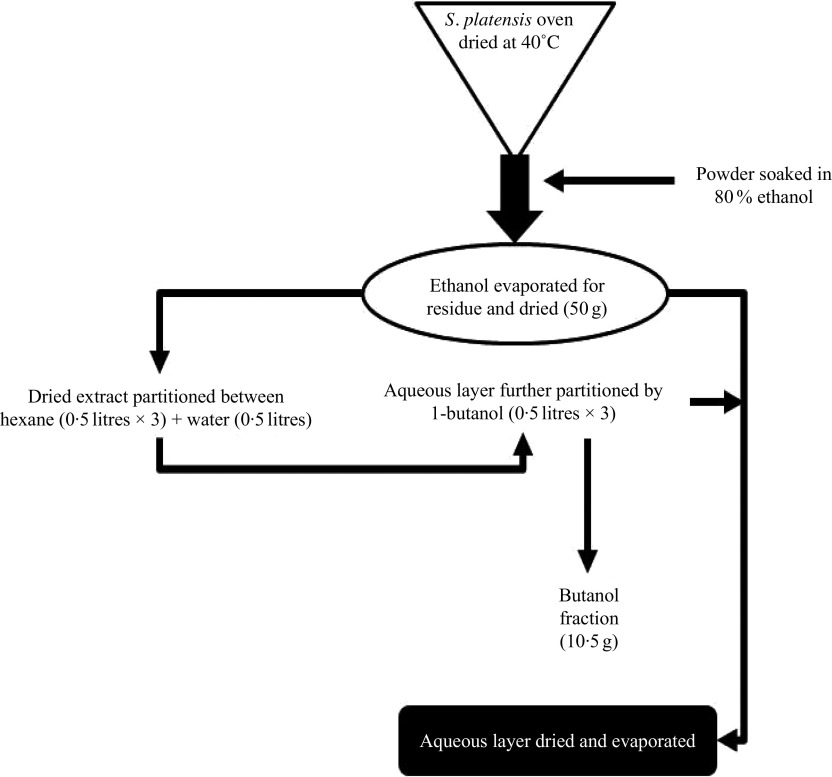
Schematic diagram of preparation of ethanol extract and butanol partition fraction of *Spirulina platensis*.

*S. platensis* was purchased from Bangladesh Council of Scientific and Industrial Research, Dhaka, Bangladesh and was authenticated by a botanical taxonomist. A voucher specimen was deposited in the Bangladesh National Herbarium (Mirpur, Dhaka). The whole plants were dried at 40°C (oven) and processed into fine powder by a cyclotec grinding machine. The powder (2 kg) was extracted with 80 % ethanol (10 litres) in a conical flask and put into an orbital shaker (550 rpm, 48–72 h). The extract was filtered using a Whatman filter paper, and ethanol was removed using a rotary evaporator (Fig. 1). A Varian 801 LY-3-TT freeze dryer (Varian) was used to freeze-dry the extract which was stored at 4°C until used.

### Preparation of butanol fraction of *Spirulina platensis*

According to the previously described method^(26)^, the ethanol extract (50 g) was partitioned into hexane (0·5 litres × 3) and water (0·5 litres). Hexane fraction was separated, and soluble material (15 g) was isolated after evaporation to dryness. The water layer was additionally partitioned using 1-butanol (0·5 litres × 3), and soluble materials of 1-butanol (10·5 g) were obtained after evaporation to dryness. The leftover watery portion was further concentrated using a rotary evaporator, and the end product was dried (20 g) using a freeze dryer and stored in a refrigerator at 4°C until used (Fig. 1). The ethanol extract and butanol fraction were analysed for bioactivity in the present studies.

### Insulin secretion from isolated islets and *β*-cell lines

The effects of *S. platensis* on insulin release from BRIN-BD11 cells and isolated mouse islets were assessed as described previously^(27)^. A range of concentrations of plant extract or butanol fraction or known modulators of insulin secretion were incubated with BRIN-BD11 cells in the presence or absence of glucose (1·1, 5·6 or 16·7 mm) during 40- or 20-min incubation at 37°C. Islets were isolated from the pancreas of NIH Swiss mice^(27)^. Groups of ten islets were cultured for 24–48 h in Roswell Park Memorial Institute (RPMI) media prior to pre-incubation in Krebs-Ringer bicarbonate (KRB) buffer at 1·4 mm glucose for 60 min. Test incubations were performed in the presence of 16·7 mm glucose for 60 min. After incubation, the supernatants were collected and stored at –20°C until analysed by insulin radioimmunoassay^(28)^.

### Insulin secretion from perfused pancreas

Long-Evans male rats (180–250 g body weight (b.w.)) were anaesthetised with sodium-pentobarbital (50 mg/kg, intraperitoneal), and the pancreas was isolated and perfused at 37°C according to the method of Giroix *et al.* ^(29)^. KRB buffer supplemented with 1·25 g/l bovine serum albumin and 40 g/l dextran T70 and 2·8- or 11·2-mm glucose. A mixture of O_2_–CO_2_ (95:5) was continuously used to gas the perfusate. The composition of the perfusate was changed after the first 20 min of equilibration as indicated in Fig. 3. Samples were stored at –20°C prior to measurement of insulin using ELISA kits supplied by Crystal Chem.

**Fig. 2.**
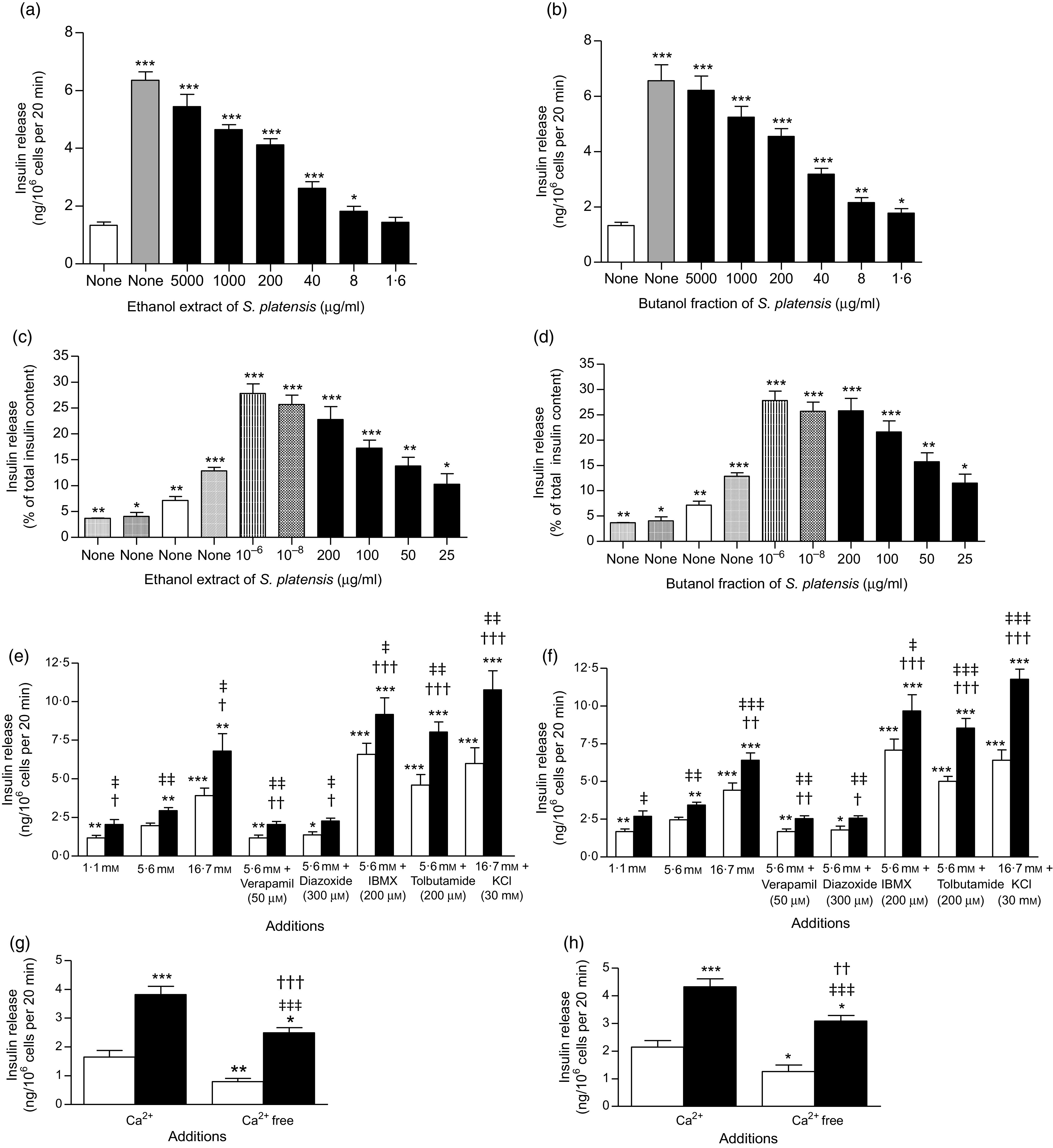
Effects of ethanol extract and butanol fraction of *Spirulina platensis* on insulin release from (a and b) BRIN-BD11 cells, (c and d) islets of Langerhans and (e–h) BRIN-BD11 cells in the presence of established stimulators or inhibitors of insulin secretion. Values are means with their standard errors, *n* 8 and 4 for insulin release. * *P* < 0·05, ** *P* < 0·01, *** *P* < 0·001 compared with 5·6 and 16·7 mm glucose alone. † *P* < 0·05, †† *P* < 0·01 and ††† *P* < 0·001 compared with 5·6 mm glucose in the presence of the extract or fraction. ‡ *P* < 0·05, ‡‡ *P* < 0·001, ‡‡‡ *P* < 0·001 compared with respective incubation in the absence of the extract or fraction. (a) 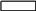, 5·6 mm glucose; 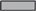, 5·6 mm glucose + 10 mm alanine; 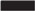, 5·6 mm glucose + ethanol extract (μg/ml). (b) 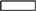, 5·6 mm glucose; 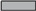, 5·6 mm glucose + 10 mm alanine; 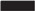, 5·6 mm glucose + butanol fraction (μg/ml). (c) 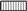, 1·4 mm glucose; 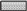, 5·6 mm glucose; 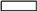, 16·7 mm glucose; 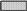, 10 mm alanine; 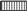, 16·7 mm glucose + glucagon-like peptide 1 (m); 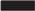, 16·7 mm glucose + ethanol extract (μg/ml). (d) 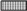, 1·4 mm glucose; 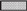, 5·6 mm glucose; 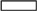, 16·7 mm glucose; 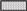, 10 mm alanine; 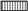, 16·7 mm glucose + glucagon-like peptide 1 (m); 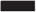, 16·7 mm glucose + butanol fraction (μg/ml). (e) 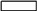, Glucose alone; 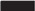, glucose + ethanol extract (40 µg/ml). (f) 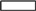, Glucose alone; 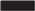, glucose + butanol fraction (40 µg/ml). (g) 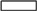, Glucose (5·6 mm); 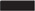, glucose (5·6 mm) + ethanol extract (40 µg/ml). (h) 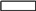, Glucose (5·6 mm); 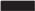, glucose (5·6 mm) + butanol fraction (40 µg/ml).

**Fig. 3.**
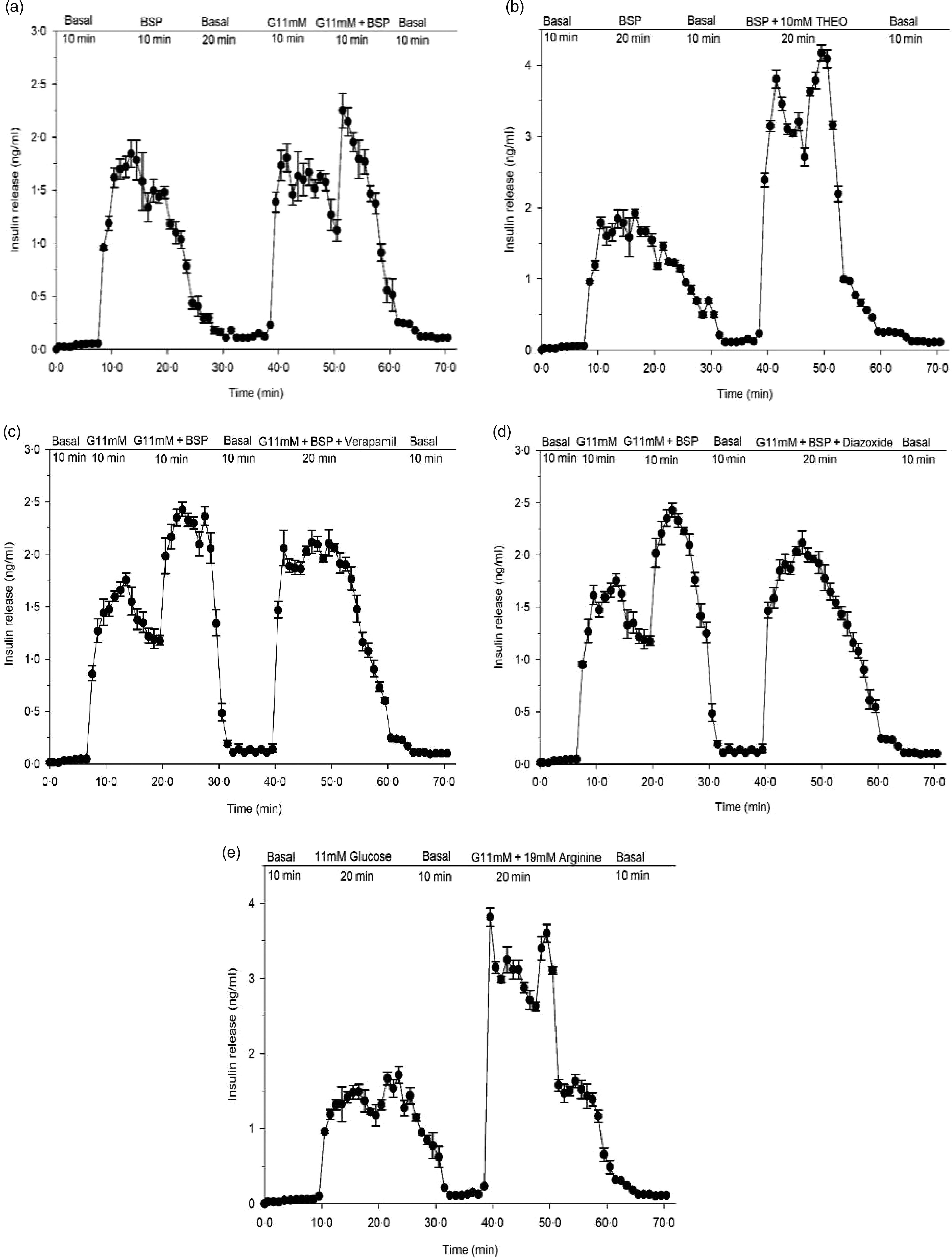
Effects of butanol fraction of *Spirulina platensis* on insulin release from perfused rat pancreas in the (a) absence or (b) presence of theophylline (10 mm), (c) verapamil (50 µm) and (d) diazoxide (8 mm) at 11 mm glucose and (e) control group: arginine (19 mm) alone. Values are means with their standard errors, *n* 4. Pancreas was perfused (1 ml/min) with butanol fraction of *S. platensis* at a dose of 5 mg/ml in the presence or absence of theophylline (10 mm), verapamil (50 µm) and diazoxide (8 mm) at 11 mm glucose and control group: arginine (19 mm) alone. The glucose concentration was raised from the basal level of 2·8 mm (basal) to 11 mm. G, glucose, THEO, theophylline; BSP, butanol fraction of *S. platensis.*

### Membrane potential and intracellular calcium ([Ca^2+^]_i_)

The effects of *S. platensis* on membrane potential and intracellular Ca were measured using BRIN-BD11 cell monolayers as previously described^(30,31)^. Cells were seeded onto ninety-six-well plates (black-walled, clear-bottomed microplates, Greiner) and washed with KRB buffer prior to the addition of FLEX assay reagents (Molecular Devices). Effects of introducing extract or butanol fraction and other reagents were monitored fluorometrically using Flex Station 3 (Molecular Devices) at wavelengths of 450 and 525 nm, respectively.

### Dipeptidyl peptidase IV activity

Effects of *S. platensis* on DPP-IV enzyme activity were determined using a fluorometric method. Enzyme activity was determined using ninety-six-well black-walled, clear-bottomed microplates (Greiner) containing 8 mU/ml of DPP-IV enzyme and 200 µm of substrate (Gly-Pro-AMC) as described previously^(32)^. Flex Station 3 (Molecular Devices) was used to measure the changes in fluorescence with an excitation and emission at 370 and 440 nm with 2·5 nm slit width, respectively.

### Induction of experimental diabetes

Long-Evans male rats (150–200 g) were purchased from the International Center for Diarrheal Disease Research, Bangladesh. Standard environmental conditions with temperature of 22 (sem 5)°C, relative humidity of 55–65 % and 12 h light–12 h dark cycle were maintained. Food and fresh water were supplied *ad libitum*. The composition of the pelleted diet (metabolisable energy of 11·8 MJ/kg per 2820 kcal/kg) was described previously^(33)^. T2DM was induced in neonatal rats at 2 d of age by a single intraperitoneal injection of streptozotocin (90 mg/kg b.w.). T2DM rats were selected for the experiments at 12 weeks of age after an oral glucose tolerance test. Animals exhibiting blood glucose levels of 8–12 mmol/l were chosen as type 2 diabetes rats. The ‘Principles of Laboratory Animals Care’ (National Institutes of Health Publication no. 86-23, revised 1985) and the UK Animals Scientific Procedures Act 1986 were followed.

### Residual gut sucrose content

The effects of *S. platensis* on sucrose digestion and absorption from the GI tract were assessed after the oral administration of sucrose solution (2·5 g/kg b.w.) to 24-h fasted T2DM rats with or without butanol fraction of *S. platensis* (250 mg/kg, b.w.). Blood samples were collected from the tip of the tail prior to and after 30, 60, 120 and 240 min for glucose analysis. To measure the unabsorbed sucrose content of the GI tract, rats were killed at the same time points and the tract was excised and divided into six parts: the stomach, the upper (20 cm), middle and lower (20 cm) of the small intestine, the caecum and the large intestine. Each segment was washed with acidified ice-cold saline and centrifuged for 10 min at 3000 rpm (1000 ***g***). The resulting supernatant was boiled for 2 h to hydrolyse the sucrose, and pH was adjusted (7·0–7·4) by adding NaOH. The concentration of serum glucose and the total amount of glucose liberated from GI tract were determined^(34)^.

### Intestinal glucose absorption

The effects of *S. platensis* on intestinal glucose absorption were determined using an *in situ* intestinal perfusion technique^(35)^. Non-diabetic rats were fasted for 36 h followed by induction of anaesthesia with sodium pentobarbital (50 g/kg b.w.). Butanol fraction of *S. platensis* (5 mg/ml equal to 0·25 g/kg) dissolved in KRB buffer containing glucose (54 g/l) was infused via the rat pylori, and perfusate was collected at the end of ileum. The control group was treated with KRB buffer only in the presence of glucose. Perfusion of intestine was carried out at a constant rate of 0·5 ml/min for 30 min at 37°C. The results were expressed as the percentage of glucose absorbed, measured from the amount of glucose in solution before and after the perfusion of intestine.

### Intestinal disaccharidase activity and gastrointestinal motility

Intestinal disaccharidase enzyme activity was determined as described previously^(33)^. Non-diabetic rats were fasted for 20 h followed by the oral administration of *S. platensis* (250 mg/kg) or water alone. The rats were killed after 1 h, and the small intestine was collected, cut longitudinally and rinsed with ice-cold saline. Volume was made up to 10 ml by adding saline (0·9 % NaCl), and the tissue was homogenised. Aliquots of the homogenate were incubated at 37°C in a 40 mm sucrose solution for 60 min. Disaccharidase enzyme activity was measured as µmol/mg protein per h. Acarbose (200 mg/kg), an established disaccharidase enzyme inhibitor, was used as a control. GI motility was measured according to the method of Chatterjee^(36)^. The butanol fraction of *S. platensis* (250 mg/kg b.w.) or water (10 ml/kg) was administered orally to non-diabetic rats. One hour later, a suspension of BaSO_4_ milk (10 % BaSO_4_ and 0·5 % carboxymethyl cellulose; w/v) was administered orally. Rats of both groups were killed after 15 min of administration of BaSO_4_. The length travelled by BaSO_4_ milk was calculated and expressed as the percentage of the total distance of the small intestine (pylorus to the ileo-caecal junction). The established drugs: Loperamide (5 mg/kg b.w.) and Sennoside (10 mg/kg b.w.) were used as positive controls.

### Glucose tolerance and chronic effects in type 2 diabetic rats

The effects of butanol fraction of *S. platensis* on oral glucose tolerance were measured after fasting the T2DM rats for 12 h. Blood samples were obtained from the cut tip of the tail at given time points (0, 30, 60, 120 and 180 min) before and after oral administration of glucose (2·5 g/kg b.w.) with (Treated group) or without (Control group) *S. platensis* (250 mg/kg). The long-term effects of *S. platensis* on glucose homoeostasis in T2DM rats were measured by twice daily administration (oral gavage) of butanol fraction (250 mg/kg) for 28 d. Control rats received oral administration of water alone. Blood samples were collected, and serum was separated by centrifugation and stored at –20°C until measurement of glucose, insulin and lipid profiles. Glucose and insulin were measured via the glucose oxidase-phenol amino phenazone (GOD-PAP) method (glucose kit, Randox™) and rat insulin ELISA Kit (Crystal Chem™), respectively.

### Effects of *Spirulina platensis* on liver glycogen content

The effects of *Spirulina platensis* treatment for 28 d on liver glycogen content were determined as previously described^(37)^. Briefly, the weight of liver was measured and finely homogenised with 10 ml of 5 % trichloroacetic acid. The precipitated proteins were filtered, and glycogen content was analysed from the clear filtrate. One millilitre of the filtrate was mixed with 2 ml of 10 N KOH and boiled for 1 h at 100°C. After cooling, 1 ml of glacial acetic acid was added and the solution was made up to 10 ml by adding deionised water. Then, 1 ml of this solution was mixed on ice with 2 ml of anthrone solution (100 mg anthrone dissolved in 50 ml of concentrated H_2_SO_4_); this mixture was boiled for additional 10 min at 100°C and then cooled. Aliquots were taken in a microplate reader, and the absorbance was measured at 490 nm.

### Insulin content in pancreases

Rats were killed after 28 d treatment with *S*. *platensis* (250 mg/kg), and the pancreatic tissues were dissected, weighed, homogenised and extracted in (10 ml) acid alcohol solution (23·5 % distilled water, 75 % ethanol and 1·5 % HCl 12 mm). After centrifugation, the supernatant samples were stored at –20°C. Pancreatic insulin level was determined using rat insulin ELISA Kit (Crystal Chem^TM^).

### Chemical characterisation by reversed-phase HPLC

Crude extract was re-dissolved in solvent A (0·12 % (v/v) trifluoroacetic acid (TFA)–water) and purified by reversed-phase HPLC. The prepared crude extract solution was injected in to a Vydac 218TP1022 (C-18) reversed-phase HPLC column (Grace) equilibrated with 0·12 % (v/v) TFA/water at a flow rate of 1·0 ml/min. The concentration of acetonitrile within the eluting solvent was expanded using linear gradients 0 to 20 % over 10 min and to 70 % over a period of 25 min. The wavelengths of 254 and 360 nm were used to measure the absorbance.

### Statistical analysis

Statistical analyses were performed by using SPSS for windows (version 20). The results are represented as mean values with their standard errors. Data were analysed using repeated-measures ANOVA followed by Dunnett’s adjustment and unpaired *t* test where applicable. *P*<0·05 was considered as the level of significance.

## Results

### Effects of *Spirulina platensis* on insulin secretion from clonal pancreatic *β*-cells (BRIN BD11)

Fig. 2(a) and (b) shows the effects of ethanol extract and butanol fraction of *S. platensis* on insulin secretion from BRIN-BD11 cells. Alanine (10 mm) was used as a positive control. Both extract and fraction (1·6–5000 µg/ml) stimulated insulin release concentrations dependently compared with control (5·6 mm glucose). Higher concentrations (1000–5000 µg/ml) also induced insulin release but were associated with decreased cell viability. Further tests revealed that the insulinotropic effects of a non-toxic dose of *S. platensis* (40 µg/ml) were significantly enhanced in the presence of 16·7 mm glucose (*P* < 0·001), isobutyl-methyl xanthine (*P* < 0·001) and tolbutamide (*P* < 0·001, Fig. 2(e) and (f)). The phosphodiesterase inhibitor isobutyl-methyl xanthine and tolbutamide were used to modulate the glucose-induced cAMP production and insulin secretion. In contrast, the effects of both ethanol extract and butanol fraction of *S. platensis* were inhibited by 40–50 % by diazoxide (*P* < 0·05) and verapamil (*P* < 0·01, Fig. 2(e) and (f)). Both diazoxide, a K_ATP_ channel opener and verapamil, voltage-gated Ca channel blocker inhibited pancreatic insulin secretion by reducing intracellular Ca^2+^ influx. The ethanol extract and butanol fraction also maintained an ability to increase insulin secretion in cells depolarised with 30 mm KCl (*P* < 0·001, Fig. 2(e) and (f)). Absence of Ca^2+^ (Fig. 2(g) and (h)) significantly inhibited but did not totally abolish insulin secretion induced by *S. platensis.*

### Effects of *Spirulina platensis* on insulin secretion from isolated islets

The ethanol extract and butanol fraction significantly increased insulin secretion from isolated mouse islets compared with 16·7 mm glucose alone (*P* < 0·05–*P* < 0·001; Fig. 2(c) and (d)). Increasing the concentrations from 25 to 200 µg/ml resulted in 2- and 2·5-fold increase in insulin release compared with control (16·7 mm glucose); glucagon-like peptide 1 (10^−6^ and 10^−8^ m) and alanine (10 mm) were used in this experiment as positive control (Fig. 2(c) and (d)).

### Effects of *Spirulina platensis* on insulin secretion from perfused pancreas

In a pilot study, the pancreas retained insulin secretory capacity during 70 min with exposure to glucose and arginine (Fig. 3(e)). Butanol fraction of *S. platensis* produced a significant (*P* < 0·001) biphasic increase in insulin release with a 20-fold elevation above the basal level (2·8 mm) (Fig. 3(a)). Subsequent exposure for 10 min to 11 mm glucose caused a sharp rise of insulin release from the basal level of 0·05–0·01 ng/ml to a peak of 2·4–1·5 ng/ml (*P* < 0·001). After adding butanol fraction to 11 mm glucose, a further enhancement in insulin release was noted (Fig. 3(a)), which opposed the decline in insulin release under the continuous exposure to 11 mm glucose alone. Theophylline (10 mm) is a methylxanthine derivative that inhibits cyclic nucleotide phosphodiesterase and increases the intracellular cyclic 3′,5′-AMP level. Addition of theophylline (10 mm) to butanol fraction increased insulin secretion further (Fig. 3(b)). As shown in Fig. 3(c) and (d), perfusion in the presence of verapamil and diazoxide at 11 mm glucose decreased insulin releasing activity of the butanol fraction by 20–30 %.

### Effects of *Spirulina platensis* on membrane potential and intracellular calcium ([Ca^2+^]_i_) in the clonal BRIN-BD11 cell line

**Fig. 4.**
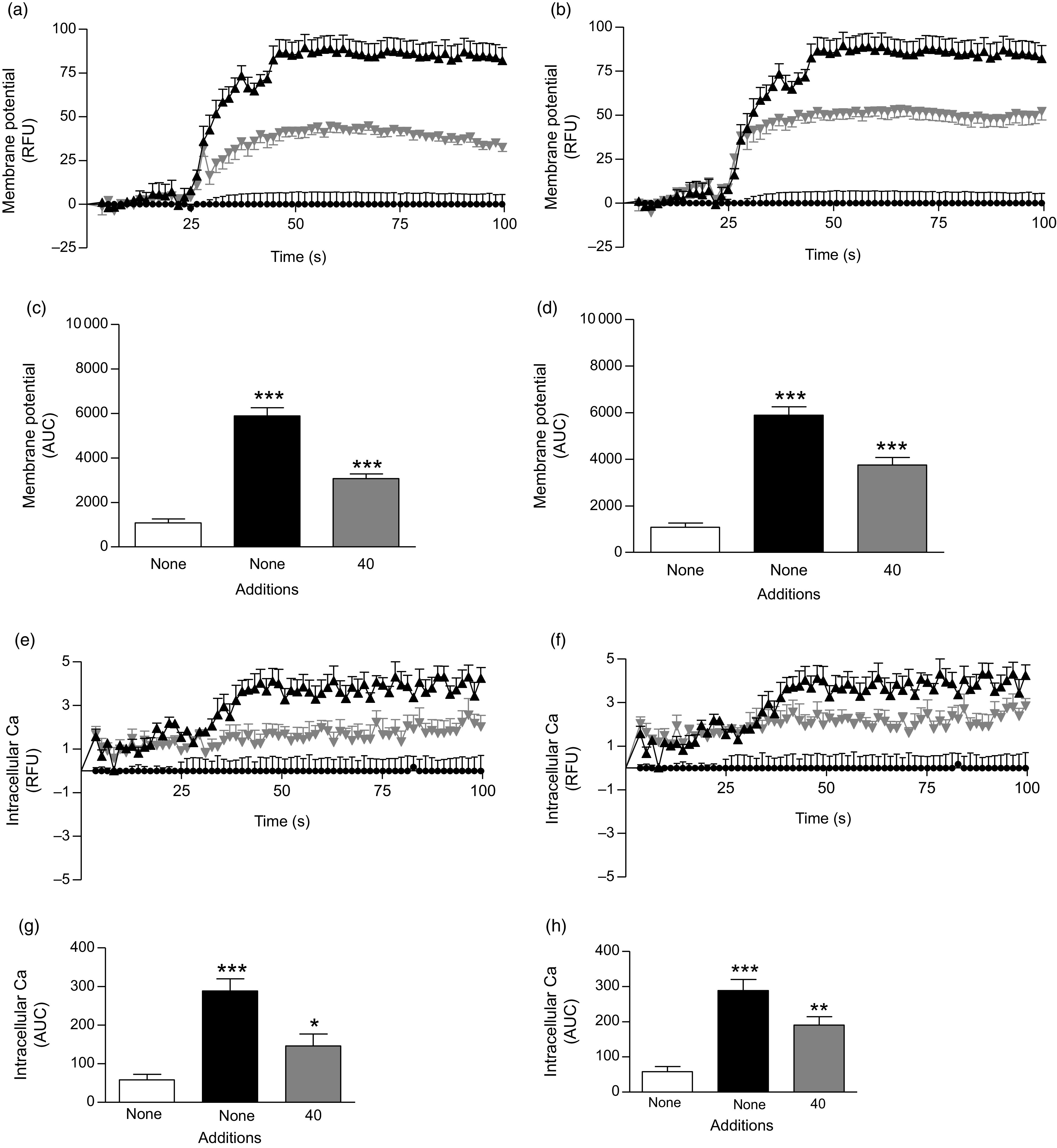
Effects of ethanol extract and butanol fraction of *Spirulina platensis* on (a–d) membrane potential and (e–h) intracellular calcium in BRIN-BD11 cells expressed as relative fluorescence units (RFU) and respective AUC. Values are means with their standard errors, *n* 6 for membrane potential and intracellular calcium. *** *P* < 0·001 compared with 5·6 mm glucose alone. (a and c) 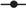, 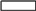, 5·6 mm glucose; 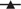, 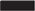, 5·6 mm glucose + KCl (30 mm); 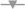, 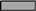, 5·6 mm glucose + ethanol extract (40 µg/ml). (b and d) 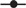, 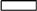, 5·6 mm glucose; 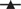, 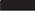, 5·6 mm glucose + KCl (30 mm); 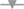, 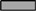, 5·6 mm glucose + butanol fraction (40 µg/ml). (e and g) 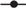, 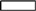, 5·6 mm glucose; 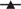, 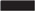, 5·6 mm glucose + 10 mm alanine; 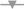, 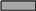, 5·6 mm glucose + ethanol extract (40 µg/ml). (f and h) 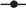, 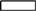, 5·6 mm glucose; 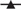, 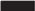, 5·6 mm glucose + 10 mm alanine; 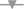, 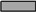, 5·6 mm glucose + butanol fraction (40 µg/ml).

The ethanol extract and butanol fraction of *S. platensis* increased membrane potential and intracellular Ca ([Ca^2+^]_i_) at the presence of 5·6 mm glucose (*P* < 0·05; Fig. 4(a–h)) as compared with control (5·6 mm glucose alone). KCl (30 mm) and alanine (10 mm) were used as positive controls in this experiment.

### Effects of *Spirulina platensis* on dipeptidyl peptidase IV activity *in vitro*

**Fig. 5.**
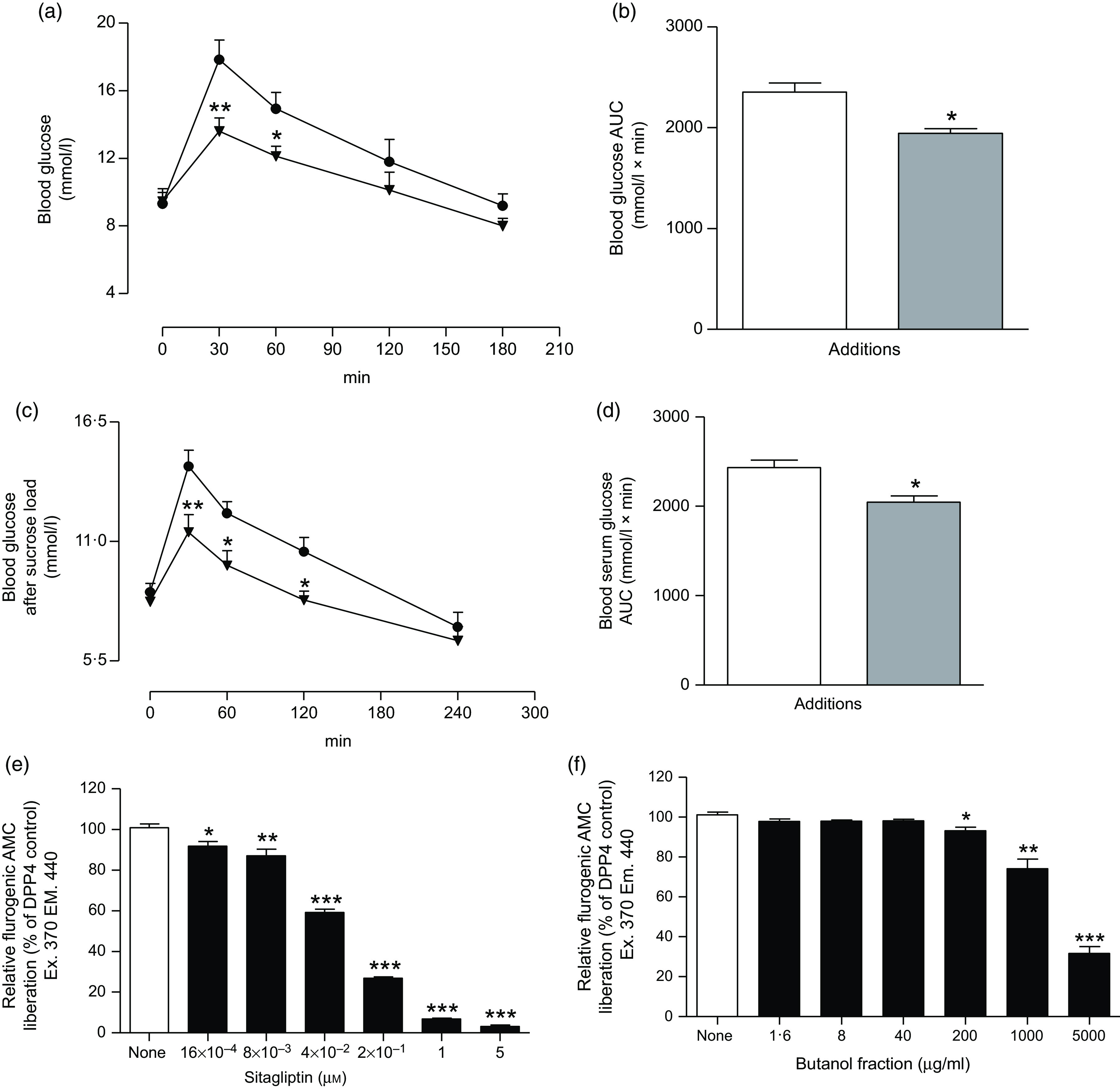
Effects of butanol fraction of *Spirulina platensis* on (a and b) glucose tolerance (GTT), (c and d) serum glucose after sucrose load (SGASL) in type 2 diabetic rats and (e and f) DPP-IV activity *in vitro*. Rats were fasted for 12 and 24 h and administered glucose or sucrose solution (2·5 g/kg body weight) by oral administration in presence or absence of butanol fraction of *S. platensis* (250 mg/kg body weight). Sitagliptin was used as established DPP-IV inhibitor. Values are means with their standard errors represented by vertical bars (*n* 6, for GTT and SGASL and *n* 3 for DPP-IV). * *P* < 0·05, ** *P* < 0·01 and *** *P* < 0·001 compared with control. (a and c) 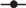, Control; 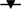, butanol fraction (250 mg/kg). (b and d) 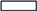, Control; 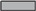, butanol fraction (250 mg/kg). (e) 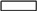, Control; 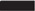, sitagliptin (µm). (f) 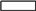, Control; 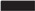, butanol fraction (µg/ml).

The butanol fraction significantly (*P* < 0·05, *P* < 0·01 and *P* < 0·001) inhibited the liberation of 7-amino-4-methylcoumarin, by 7–70 % at concentrations of 200–5000 µg/ml (Fig. 5(f)). Sitagliptin, as an established DPP-IV inhibitor, reduced DPP-IV activity by 10–97 % (*P* < 0·05, *P* < 0·01 and *P* < 0·001; Fig. 5(e)) at concentrations between 16 × 10^−4^ to 5 µm.

### Effects of *Spirulina platensis* on unabsorbed sucrose content in the gut

**Fig. 6.**
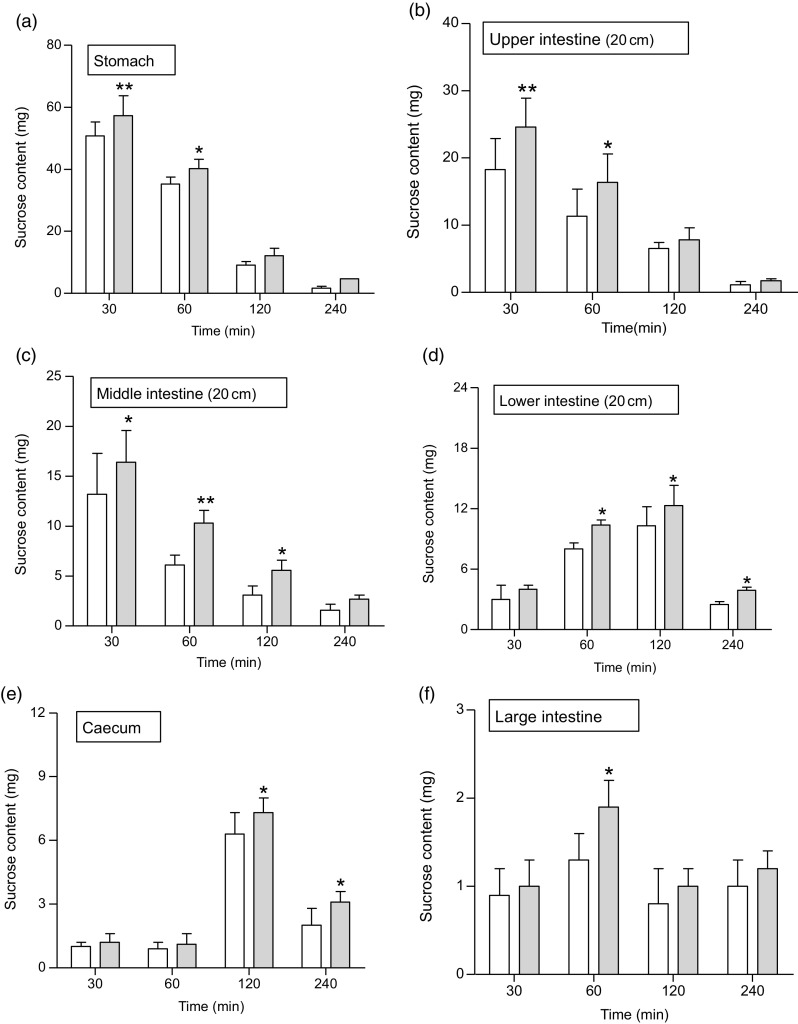
Effects of butanol fraction of *Spirulina platensis* on (a–f) gastrointestinal sucrose content after oral sucrose loading in type 2 diabetic rats. Type 2 diabetic rats were fasted for 24 h prior to the oral administration of sucrose solution (2·5 g/kg body weight) in the presence (treated group) or absence of (control group) butanol fraction of *S. platensis* (250 mg/kg body weight). Values are means with their standard errors represented by vertical bars (*n* 6). * *P* < 0·05 and ** *P* < 0·01 compared with type 2 diabetic control rats. (a–f) 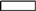, Control; 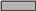, butanol fraction (250 mg/kg).

At 1 h following sucrose load (2·5 g/kg b.w.), a significant (*P* < 0·05–0·01) amount of sucrose was measured in the stomach and the upper, middle, lower and large intestine (Fig. 6(a–f)). An extensive (*P* < 0·01) amount of sucrose found in the stomach, upper and middle intestine at 30 min, while at 2 h (*P* < 0·05), increased amounts of unabsorbed sucrose were measured in caecum and lower intestine. The administration of butanol fraction (250 mg/kg b.w.) with sucrose reduced sucrose absorption significantly (*P* < 0·01) after 30 min and up to 2 h (Fig. 6). At 4 h, sucrose content was almost nil throughout the GI tract, although a small fraction remained in the caecum and lower intestine, indicating rapid hydrolysis and absorption of sucrose in the upper part of the intestine (Fig. 6).

### Effects of *Spirulina platensis* on intestinal glucose absorption

**Fig. 7.**
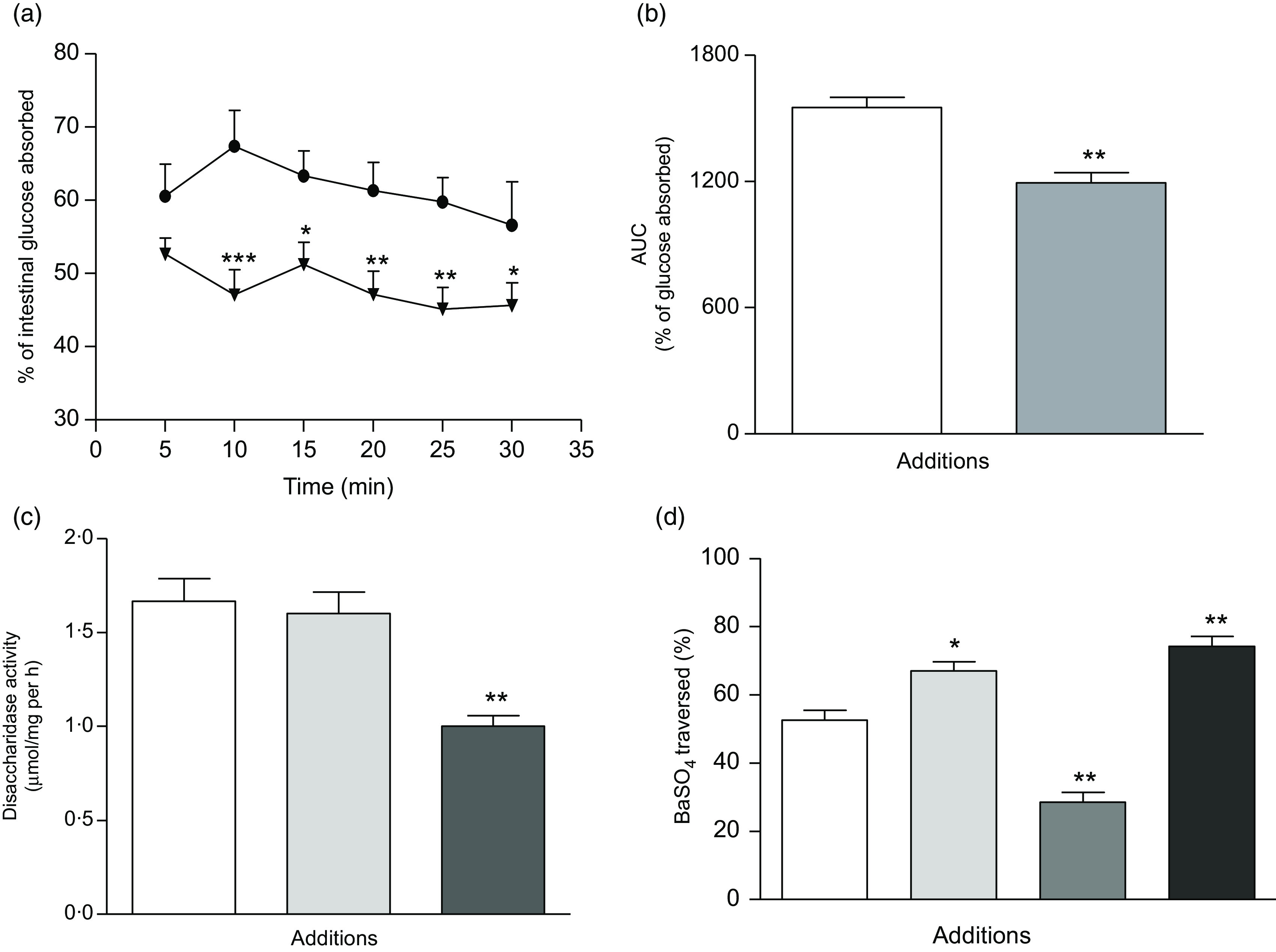
Effects of butanol fraction of *Spirulina platensis* on (a and b) intestinal glucose absorption, (c) disaccharidase enzyme activity and (d) gastrointestinal motility (by BaSO_4_ traversed) in non-diabetic rats. Rats were fasted for 36 h, and intestine was perfused with glucose (54 g/l) in the presence (treated group) or absence of (control group) butanol fraction of *S. platensis* (10 mg/ml). BaSO_4_ was administered at 60 min following oral feeding of *S. platensis*. Acarbose (200 mg/kg); and loperamide (5 mg/kg) and sennoside (10 mg/kg) were used as positive controls for determinations of disaccharidase activity and gastrointestinal motility, respectively. Values are means with their standard errors represented by vertical bars (*n* 8). * *P* < 0·05, ** *P* < 0·01 and *** *P* < 0·001 compared with controls. (a) 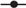, Control; 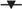, butanol fraction (250 mg/kg). (b) 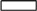, Control; 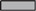, butanol fraction (250 mg/kg). (c) 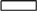, Control; 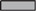, butanol fraction (250 mg/kg); 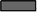, acarbose (200 mg/kg). (d) 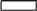, Control; 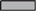, butanol fraction (250 mg/kg); 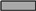, loperamide (5 mg/kg); 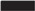, sennoside (10 mg/kg).

During perfusion, the extent of glucose absorption in the intestine was almost consistent over 30 min. However, supplementation with butanol fraction significantly decreased intestinal glucose absorption (*P* < 0·05–*P* < 0·01) as illustrated in Fig. 7(a) and (b).

### Effects of *Spirulina platensis* on intestinal disaccharidase activity and gastrointestinal motility

Butanol fraction of *S. platensis* (250 mg/kg b.w.) did not affect disaccharidase enzyme activity (Fig. 7(c)). However, this fraction significantly promoted gastrointestinal motility (*P* < 0·05, Fig. 7(d)).

### Acute and chronic effects of *Spirulina platensis* on glucose homoeostasis in type 2 diabetic rats

Oral administration of butanol fraction of *S. platensis* (250 mg/kg b.w.) together with glucose (2·5 g/kg b.w.) improved glucose tolerance at 30 and 60 min (*P* < 0·01–*P* < 0·05) in T2DM rats (Fig. 5(a) and (b)). In addition, *S. platensis* (250 mg/kg b.w.) treatment lowered serum glucose significantly after sucrose load (*P* < 0·05; Fig. 5(c) and (d)) at 60 and 120 min in T2DM rats.

Furthermore, twice daily oral administration of the butanol fraction of *S. platensis* (250 mg/kg b.w.) for 28 d significantly lowered serum glucose levels (*P* < 0·05) and increased serum insulin level (*P* < 0·05) compared with controls (Table 1). Pancreatic insulin and liver glycogen were increased (*P* < 0·05) as shown in Table 1. Measurement of lipid profile showed that the butanol fraction of *S. platensis* (250 mg/kg b.w.) increased HDL, while the LDL and total cholesterol were significantly decreased (*P* < 0·05–0·01; Table 1).

**Table 1.** Long-term effects of the butanol fraction of *Spirulina platensis* on blood glucose, plasma insulin, pancreatic insulin content and other parameters in type 2 diabetic rats after a 28-d study† (*n* 8) (Mean values with their standard errors)

Parameters	0 d				28 d			
	Control		Butanol fraction		Control		Butanol fraction	
	Mean	sem	Mean	sem	Mean	sem	Mean	sem
Glucose (mmol/l)	9·71	1·41	9·53	1·27	9·67	1·45	7·31*	1·17
Plasma insulin (ng/ml)	0·47	0·19	0·48	0·29	0·46	0·18	0·67*	0·15
Pancreatic insulin (nmol/g)	–		–		0·81	0·15	1·10*	0·11
Liver glycogen (g/100 g)	–		–		1·45	0·22	2·26*	0·30
Total cholesterol (mmol/l)	–		–		3·53	0·20	2·60*	0·12
HDL (mmol/l)	–		–		2·41	0·15	3·15*	0·12
LDL (mmol/l)	–		–		2·05	0·23	0·75**	0·16

* *P* < 0·05 and ** *P* < 0·01 compared with type 2 diabetic control rats.

† Butanol fraction of *S. platensis* (250 mg/kg body weight) or only saline (control) was administered orally to rats for 28 d.

### Chemical characterisation by reversed-phase HPLC

**Fig. 8.**
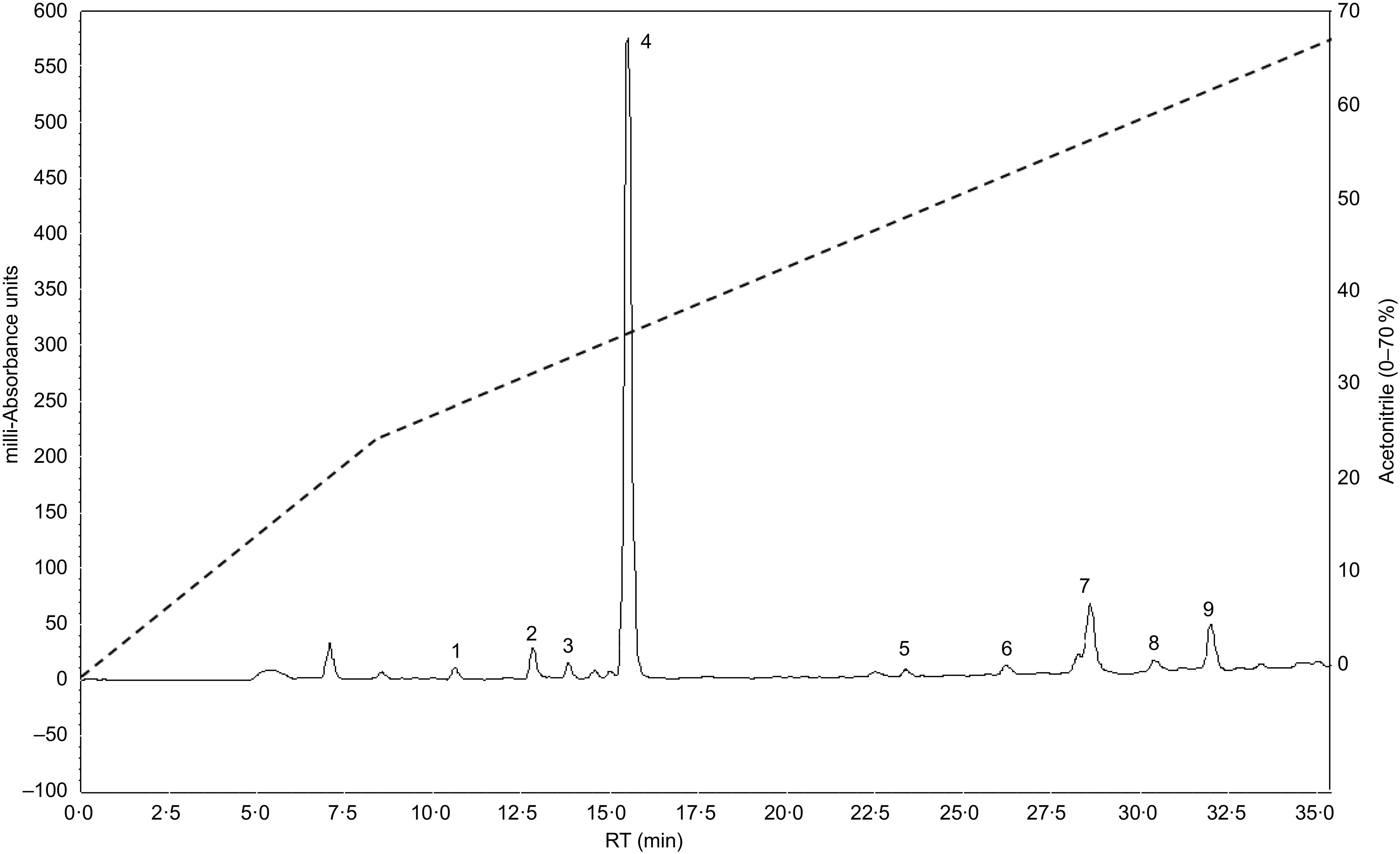
Representative HPLC profile of butanol fraction of *Spirulina platensis* using analytical C-18 column over the period of acetonitrile. The column was equilibrated with 0·1 % (v/v) trifluoroacetic acid/water at flow rate of 1·0 ml/min. The concentration of the eluting solution was raised using linear gradients from 0 to 20 % acetonitrile over 10 min, to 70 % over 25 min. Details of peaks corresponding to butanol fraction are presented in the chromatogram. UV detection was set at 254 and 360 nm, and 1 mg/ml sample was injected each run. Peaks 1–9 of unknown compounds were detected at different retention times (RT).

The phytochemical screening of butanol fraction by HPLC (Fig. 8) revealed the presence of *β*-carotene, catechin and some other previously identified compounds in *S. platensis* such as zeaxanthin, astaxanthin, *p*-coumaric acid and apigenin^(38,39)^. The concentrations of nutritional chemical characterisation of *S. platensis* are listed in Table 2.

**Table 2.** Major compounds identified by reversed-phase-HPLC of the butanol fraction *of Spirulina platensis*

Peaks	Compounds	RT (min)	Concentration (µg/ml)
1	Siphonein	10·3	20·6
2	Zeaxanthin	12·6	30·5
3	Astaxanthin	13·0	17·8
4	Unidentified carotenoids	15·3	122·4
5	Catechin	23·5	15·6
6	Pheophytin A	26·2	15·6
7	*p*-Coumaric acid	29·0	51·3
8	*β*-Carotene	30·8	22·1
9	Apigenin	32·4	29·7

RT, retention time.

## Discussion

*S. platensis* has been reported recently to exhibit antihyperglycaemic effects, but the mechanism of action was not elucidated^(23–25)^. This study has examined the insulinotropic effects of *S. platensis* using the perfused rat pancreas, isolated mouse islets and the clonal BRIN-BD11 *β*-cell line. The results suggest that the anti-hyperglycaemic effects are partly mediated through stimulating the insulin secretion from pancreatic *β*-cells^(27)^, which is further supported by observation of incremental increases in serum and pancreatic insulin after 28 d of chronic treatment.

Both the ethanol extract and butanol fraction dose-dependently enhanced insulin release from isolated islets and clonal BRIN-BD11 cells. The butanol fraction was more insulinotropic than the ethanol extract. Likewise, the butanol fraction exerted substantial insulin release from perfused rat pancreas. Non-toxic concentrations of *S. platensis* were used to examine mechanisms underlying stimulation of insulin secretion in the absence and presence of known modulators of *β*-cell function. Sulfonylureas are known to act by closing K_ATP_ channels, depolarising the plasma membrane, as induced by 30 mm KCl, and stimulating Ca^2+^ entry by activation of voltage-dependent Ca channels^(26,40)^. *S. platensis* stimulated insulin release enhanced by tolbutamide and KCl (30 mm), suggesting its ability to potentiate insulin secretion via other pathways such as the adenylate cyclase/cAMP or the phosphatidylinositol pathway, or as a direct effect on exocytosis^(26)^. *S. platensis* also clearly stimulated *β*-cells via its effects on Ca^2+^ ion channels. Thus, diazoxide, a K_ATP_-channel opener^(41)^, inhibited the insulin-releasing effects of both the extract and fraction. This suggests that *S. platensis* closes K_ATP_ channels to induce insulinotropic action. Furthermore, the L-type voltage-dependent Ca^2+^ channel blocker, verapamil,^(42)^ also reduced the insulin-releasing effects of *S. platensis*. This suggests a dependency on Ca^2+^ channel to induce insulin release. Similar intracellular Ca^2+^ dependency was found in BRIN-BD11 cells, where *S. platensis* increased insulin release, which was inhibited by the L-channel blocker verapamil. Furthermore, studies with BRIN BD11 cells showed *S. platensis* induced membrane depolarisation and increased [Ca^2+^]_i._

The insulin-releasing effects of *S. platensis* were also markedly increased by the phosphodiesterase inhibitors isobutyl-methyl xanthine and theophylline implicating involvement of the cAMP pathway^(43)^. Recent studies have shown that use of *S. platensis* in asthma as adjunct therapy has improved the condition significantly^(44)^. The anti-asthmatic actions have been attributed to elevation of cAMP in bronchial smooth muscle cells, promoting airway relaxation and blocking replication of smooth muscle cells^(45)^. Overall, these results indicate that the polar solvents ethanol and butanol contain active molecules of *S. platensis* that exert multiple effects on the *β*-cells mediated most importantly via ion channels.

DPP-IV is an enzyme that metabolises incretin hormones and as a result terminate the insulin-releasing and glucose-lowering actions of both glucagon-like peptide 1 and glucose‐dependent insulinotropic polypepide^(46)^. DPP-IV inhibitors have been developed to enhance endogenous incretin action and treat T2DM^(47)^. The two incretin hormones have been shown to have multiple action by increasing pancreatic insulin secretion and reducing glucagon secretion^(46)^. In the present study, *S. platensis* significantly (*P* < 0·05, *P* < 0·01 and *P* < 0·001) inhibited DPP-IV enzyme activity, indicating that butanol fraction can enhance endogenous glucagon-like peptide 1 and glucose‐dependent insulinotropic polypepide activity. A recent study has shown that flavonol glycosides from the seeds of *Lens culinaris* Medikus (Fabaceae) inhibited DPP-IV enzyme activity in the dose-dependent manner^(48)^. Therefore, it is anticipated that the same phytochemical constituents of *S. platensis* may be responsible for the observed DPP-IV inhibitory activity effect.

Administration of *S. platensis* significantly lowered blood glucose and improved glucose tolerance in T2DM rats. The butanol fraction also significantly inhibited glucose absorption during gut perfusion. As anticipated, significant amounts of unabsorbed sucrose were found in postprandial state throughout the gut. This postprandial effect may be related to the interference of intestinal glucose absorption^(33)^. The butanol fraction of *S. platensis* did not inhibit intestinal disaccharidase enzyme activity, and thus inhibition of digestion of carbohydrate is not involved in its mechanism of antihyperglycaemic action. However, increased GI motility, examined using BaSO_4_ milk, may inhibit carbohydrate absorption in the gut. Dietary fibres reduce postprandial food transit time in the GI tract^(49)^; thus, shorter time is available for the carbohydrate absorption^(50)^, and therefore postprandial hyperglycaemia is reduced. High sucrose content in the GI tract indicates reduced sucrose digestion. As a result, a significantly higher concentration of sucrose reaches to the large intestine and caecum and is excreted. *S. platensis* reduced postprandial sucrose absorption and enhanced GI motility, possibly by forming glucose–fibre complexes that reduce post prandial transit time or gastric emptying time.

A recent study using *S. platensis* reported potential protective activity against fat induced apoptosis and decreasing intestinal cholesterol absorption^(51,52)^. However, in the present chronic study, administration of butanol fraction of *S. platensis* for 28 d in type 2 diabetic rats significantly increased liver glycogen content and HDL, while it reduced LDL substantially. Interestingly, chronic treatment also enhanced plasma insulin and pancreatic insulin content in type 2 diabetic rats. Therefore, the stimulation of insulin release from *β*-cells as well as insulin action is possibly thereby co-related with the improvement of hepatic glucose uptake.

The phytochemical screening of *S. platensis* using reversed-phase HPLC revealed the presence of several phenolic acids like *p*-coumaric acid and other bioactive molecules such as pheophytin A, catechin, zeaxanthin, astaxanthin, apigenin and carotenoid pigments including *β*-carotene. This composition is in general agreement with the previous research^(38,39)^. Recent studies also claim that these compounds have antioxidant properties, trigger insulin secretion and have antihyperglycaemic activity^(53–55)^.

In conclusion, this study has shown that ethanol extract of *S. platensis* and its butanol fraction exert prominent stimulatory effects on insulin secretion from *β*-cells via physiological pathways. *In vivo* studies in T2DM rats indicate that the butanol fraction decreased blood glucose, increased gut motility, reduced glucose absorption in GIT and improved both plasma and pancreatic insulin levels. *S. platensis* contains important phytochemicals and valuable micro/macronutrients, consistent with the use of this microalgae as a prophylactic or dietary supplement in the treatment of diabetes.
